# WFUMB Review Paper. Incidental Findings in Otherwise Healthy Subjects, How to Manage: Liver

**DOI:** 10.3390/cancers16162908

**Published:** 2024-08-21

**Authors:** Roxana Șirli, Alina Popescu, Christian Jenssen, Kathleen Möller, Adrian Lim, Yi Dong, Ioan Sporea, Dieter Nürnberg, Marieke Petry, Christoph F. Dietrich

**Affiliations:** 1 Department of Gastroenterology and Hepatology, “Victor Babeș” University of Medicine and Pharmacy, 300041 Timișoara, Romania; roxanasirli@gmail.com (R.Ș.); alinamircea.popescu@gmail.com (A.P.); isporea@umft.ro (I.S.); 2 Center for Advanced Research in Gastroenterology and Hepatology, “Victor Babeș” University of Medicine and Pharmacy, 300041 Timișoara, Romania; 3 Department of Internal Medicine, Krankenhaus Märkisch Oderland GmbH, 15344 Strausberg, Germany; c.jenssen@khmol.de; 4 Brandenburg Institute for Clinical Ultrasound (BICUS) at Medical University Brandenburg “Theodor Fontane”, 16816 Neuruppin, Germany; 5 Medical Department I/Gastroenterology, SANA Hospital Lichtenberg, 10365 Berlin, Germany; k.moeller@live.de; 6 Department of Imaging, Imperial College London and Healthcare NHS Trust, London W6 8RF, UK; a.lim@imperial.ac.uk; 7 Department of Ultrasound, Xinhua Hospital Affiliated to Shanghai Jiao Tong University School of Medicine, Shanghai 200092, China; drdaisydong@hotmail.com; 8 Faculty of Medicine and Philosophy and Faculty of Health Sciences Brandenburg, 16816 Neuruppin, Germany; nuernbergdieter@gmx.de; 9 Department Allgemeine Innere Medizin (DAIM), Kliniken Hirslanden Beau Site, Salem und Permanence, 3013 Bern, Switzerland; marieke.petry@gmx.de

**Keywords:** incidental focal liver lesion, asymptomatic patients, ultrasound, contrast enhanced ultrasound (CEUS)

## Abstract

**Simple Summary:**

This review paper deals with incidentally found focal liver lesions (IFLLs) in otherwise healthy subjects, which is a frequent occurrence in daily practice. The clinical presentation and the imaging aspects play an important role in deciding whether and what further evaluation is required. In low-risk patients (i.e., those without a history of malignant or chronic liver disease or related symptoms, younger than 40 years old), more than 95% of IFLLs are benign. Shear Wave liver Elastography of the surrounding liver parenchyma should be considered to exclude liver cirrhosis and for further risk stratification. If an IFLL in a low-risk patient has a typical appearance on a B-mode ultrasound of a benign lesion, no further imaging is needed. Contrast-Enhanced Ultrasound (CEUS) should be considered as the first-line contrast imaging modality to differentiate benign from malignant IFLLs. In high-risk patients (i.e., with chronic liver disease or an oncological history), each IFLL should initially be considered as potentially malignant, and every effort should be made to confirm or exclude malignancy.

**Abstract:**

An incidental focal liver lesion (IFLL) is defined as a hepatic lesion identified in a patient imaged for an unrelated reason. They are frequently encountered in daily practice, sometimes leading to unnecessary, invasive and potentially harmful follow-up investigations. The clinical presentation and the imaging aspects play an important role in deciding if, and what further evaluation, is needed. In low-risk patients (i.e., without a history of malignant or chronic liver disease or related symptoms), especially in those younger than 40 years old, more than 95% of IFLLs are likely benign. Shear Wave liver Elastography (SWE) of the surrounding liver parenchyma should be considered to exclude liver cirrhosis and for further risk stratification. If an IFLL in a low-risk patient has a typical appearance on B-mode ultrasound of a benign lesion (e.g., simple cyst, calcification, focal fatty change, typical hemangioma), no further imaging is needed. Contrast-Enhanced Ultrasound (CEUS) should be considered as the first-line contrast imaging modality to differentiate benign from malignant IFLLs, since it has a similar accuracy to contrast-enhanced (CE)-MRI. On CEUS, hypoenhancement of a lesion in the late vascular phase is characteristic for malignancy. CE-CT should be avoided for characterizing probable benign FLL and reserved for staging once a lesion is proven malignant. In high-risk patients (i.e., with chronic liver disease or an oncological history), each IFLL should initially be considered as potentially malignant, and every effort should be made to confirm or exclude malignancy. US-guided biopsy should be considered in those with unresectable malignant lesions, particularly if the diagnosis remains unclear, or when a specific tissue diagnosis is needed.

## 1. Summary Statements and Perspectives

Incidental focal liver lesions (IFLLs) are frequently encountered in daily practice, occasionally leading to unnecessary follow-up investigations or interventions.

The diagnostic management of IFLL differs between individuals without chronic liver disease and without a history of malignancy (low risk) and patients with chronic liver disease and/or a history of malignancy (high-risk).

Shear Wave Elastography (SWE) of the surrounding liver parenchyma should be considered in patients with IFLL, to exclude liver cirrhosis/advanced fibrosis and for further risk stratification.

The vast majority of IFLLs in asymptomatic and low-risk individuals are benign.

An IFLL detected in low-risk individuals needs no further imaging if it has the typical B-mode features of a benign lesion (e.g., simple cysts, calcifications, focal fatty change, typical hemangioma).

IFLLs in patients with high-risk criteria have an increased risk of malignancy or otherwise therapeutically relevant processes and thus must be meticulously investigated by contrast-enhanced imaging. In many cases, a histological diagnosis may be required.

Contrast-Enhanced Ultrasound (CEUS) should be used as the first imaging modality to differentiate benign from malignant IFLLs.

Washout in the portal and/or late phase is a characteristic sign for an FLL (or tissue) without liver-specific portal-venous and sinusoidal vessels. This is typical for malignancies, but inflammatory lesions (e.g., inflammatory pseudotumors, granulomatous disease, immature abscesses) may also show a similar phenomenon.

Ultrasound is recommended as the most appropriate imaging modality for guiding targeted biopsies of FLLs. CEUS-guidance or fusion techniques with CT or MRI can be used to reduce the false-negative rate and to improve the diagnostic yield of a US-guided FLL biopsy.

CEUS can be used as a second-line contrast-enhanced imaging modality in cases of unclear lesions on CE-CT and/or CE-MRI.

## 2. Introduction

The World Federation for Ultrasound in Medicine and Biology (WFUMB) is dedicated to the advancement of ultrasound (US) by encouraging research, promoting international cooperation, disseminating scientific information and improving communication and understanding in the global community of US in medicine and biology. Therefore, the mission of WFUMB is to bring sustainable ultrasound programs to the underserved areas of the world and to improve global healthcare through collaboration, communication, and education (www.wfumb.org (accessed on 3 April 2024)).

WFUMB is addressing the issue of incidental findings (IF) with a series of publications entitled “Incidental imaging findings—the role of medical ultrasound” [1]. Each WFUMB position paper on IFs will follow the same template and accordingly be uniformly structured to help readers interpret the key messages [1,2,3,4,5,6,7,8,9].

This paper is intended as a guideline to all practitioners involved in liver ultrasound to try and avoid unnecessary investigations when an IF in an asymptomatic liver is found, but, at the same time, avoid the risk of missing the diagnosis of a lesion impacting on the patient’s outcome.

## 3. Incidental Findings in the Liver and Risk Constellations

*Definition*. Incidental findings (IF) on imaging studies are asymptomatic and unexpected pathology, unrelated to the presenting illness [1,10]. Therefore, an incidental focal liver lesion (IFLL) is defined as a hepatic lesion identified in a patient who has been imaged for an unrelated reason [11]. IFLLs may be cystic (fluid content), solid or a mixture of both.

*Risk constellations, pretest probability*. The clinical presentation of patients with IFLL varies considerably between a low-risk and asymptomatic individual to a patient with advanced chronic liver disease or a history of malignancy. Of course, the clinical presentation, as well as the imaging aspect, plays an important role regarding the need and recommendations for further evaluation. IFLLs are more likely benign, especially in asymptomatic subjects [12,13,14], but even in cirrhotic patients (with an increased risk of hepatocellular carcinoma—HCC) [15,16,17] and in oncological patients (with risk of metastases), benign IFLLs are frequent, especially if they are smaller than 15 mm in diameter [18,19]. The probability of a malignant lesion increases when there are deviations from the normal size, shape, and vascularization of the liver [11].

Data regarding the prevalence of IFLLs vary according to the method used for diagnosis and the population evaluated. Necropsy studies report a prevalence of IFLLs of up to 52%, which increases with age [20,21]. The detection rates of the different imaging methods are shown in Table 1.

*Work up and risk stratification.* Considering the high number of IFLLs and the potential psychological impact they generate to the patient [1], even if most IFLLs are benign, further work-up (which may include laboratory tests, contrast imaging studies and even liver biopsy in selected cases) are unavoidable in many patients. The questions are usually “Should anything be done and if so, what next?”. Several studies showed that over-diagnosis in patients with benign lesions puts them at risk for potentially dangerous and expensive procedures including biopsy and explorative surgery [33,34,35,36,37]. Further work-up can be avoided if there are no risk factors for malignancy and if the lesion has a classical appearance for a benign lesion on B-mode US or cross-sectional imaging.

The clinical history of the patient is extremely important. In patients younger than 40 years of age and without a history of malignant or chronic liver disease or any related symptoms, there is more than a 95% likelihood that the IFLL is benign [26].

### Risk Factors and “Red Flags”

*High-risk patients* for malignancy include those with a known history of malignancy, older subjects, those with liver cirrhosis or chronic liver disease and advanced liver fibrosis.

*Low-risk individuals/patients* include subjects with no clinical symptoms, no history of a malignancy (especially if younger than 40), no known metabolic or chronic liver disease, no intake of potentially harmful medications (e.g., anabolic steroids), normal biological liver tests, normal cholestasis enzymes and bilirubin, normal ultrasound appearance of the liver parenchyma and without hepatic risk factors, even after thorough anamnesis [11].

*Intermediate risk* can be defined as all patients outside either of the definitions described above. “Red flags”, or high-risk criteria prompting further work-up include the above-mentioned clinical history for high-risk patients, but also the imaging aspect of the surrounding liver parenchyma. The “red flag” criteria are summarized in Table 2.

## 4. Cross-Sectional Imaging (US, CT, MRI) for Detection and Diagnosis

If an IFLL is found in a low-risk patient on B-mode US, and it has typical features of a benign lesion (e.g., simple cyst, coarse calcification, focal fatty change in common locations, classical hyperechoic hemangioma with well-defined and sharp margins and <3 cm in diameter), then no further imaging is needed [39]. If in doubt, contrast-enhanced imaging is needed to conclusively differentiate between benign and malignant IFLLs. Contrast-Enhanced (CE) imaging techniques for the liver are based on the dual blood supply of the liver, i.e., via the hepatic artery and the portal vein. They allow the characterization of the enhancement pattern of FLLs in all vascular phases: arterial, portal-venous and late and post-vascular.

### 4.1. Conventional B-Mode Ultrasound and Advanced Ultrasound Techniques

**Detection of IFLL by transabdominal ultrasound.** Owing to the increased availability of B-mode US, IFLLs are frequently discovered, with a reported incidence ranging from 2.3–12.5% in Asian populations [12,14,25] and 19% in one European study, where most of them were characterized to be focal fatty changes (12%). Another European study showed a prevalence of benign IFLLs of 15.1% in a population of more than 45,000 subjects [22]. Interestingly, the frequency of benign IFLLs was found to be significantly higher (40.6%) in a large cohort of patients with chronic hepatitis B, compared to the general population (12.5%) [25].

For cases in which B-mode US is not sufficient to establish a conclusive diagnosis, CEUS can be performed for a definitive diagnosis, preferentially at the same examination session. The accuracy of CEUS is around 90% for the differentiation between benign and malignant FLL and around 80% for a specific diagnosis [40,41,42].

**CEUS** uses specific US contrast agents (CA) and specific US software, which helps to detect and amplify the US signal of CA. US contrast agents (UCA) are microbubbles containing an inert gas, stabilized by an outer “shell”. To avoid rapid microbubble destruction, the examination is performed with a low mechanical index [39,43]. As opposed to CA for CE-CT and CE-MRI, UCAs are strict intravascular agents and are excreted through the lungs, so that they can safely be administered in patients with renal failure [43]. The most recent EFSUMB Guidelines on CEUS recommends its use as a first-line CE imaging method in patients with renal impairment [39]. CEUS can be used as a mobile, and, therefore, point of care imaging method, is non-ionizing and has a very low risk of adverse events [39,43,44]. In reaction to liposomal-coated substances, a pseudo-anaphylactoid reaction called CARPA (complement activation-related pseudo-allergy) may occur, however it is very rare and can be treated with corticosteroids and antihistamines [43]. Prolonged heterogeneous liver enhancement has been described as a harmless phenomenon in rare cases [45].

After bolus injection, UCA quickly enhance the vascular pool, allowing characterization of the macrovasculature as well as the microvasculature, after which they slowly dissipate in 5–10 min. Most published data refer to the use of Sulfur hexafluoride gas containing microbubbles (SonoVue). More recently, Sonazoid has been introduced for liver imaging. It is phagocytosed by the Kupffer cells in the liver and spleen, where it can remain for hours, allowing IFLL examination and characterization in the post-vascular phase, past the initial 10 min.

CEUS allows the characterization of IFLLs in all vascular phases and has the advantage of allowing real-time visualization of tumor vessels and contrast uptake, with high temporal and spatial resolution, allowing the characterization of IFLLs with an accuracy similar to CE-CT and CE-MRI [46,47].

The disadvantages of CEUS are that it is a more operator-dependent method, that its performance can be impaired by patients’ characteristics (e.g., poor acoustic window, severe liver steatosis) and that it cannot visualize the entire liver at once [43]. Nevertheless, CEUS is able to differentiate benign from malignant IFLLs with approximately 90% accuracy [40,41,42,48]. Specific vascular patterns allow differentiation among various types of lesions with a similar accuracy to CE-MRI [41,46,47,49]. The WFUMB Guidelines recommend the use of CEUS as a first-line CE imaging method for the differentiation of benign versus malignant IFLL in non-cirrhotic patients, with or without a history or clinical suspicion of malignancy [39].

**Liver elastography**. US-based elastography techniques are sensitive methods to estimate liver stiffness as a marker of liver fibrosis severity [50,51]. US-based elastographic methods can be divided into Strain and Shear Wave Elastography (SWE). SWE methods are categorized into: Vibration Controlled Transient Elastography (VCTE), point SWE (pSWE) and 2D-SWE. All of the SWE methods are used in clinical practice for liver fibrosis assessment, being relevant for the diagnosis of advanced fibrosis and cirrhosis [52,53,54]. The value of SWE techniques for characterization of focal liver lesions is limited, while strain imaging is difficult to perform and unreliable [55,56,57].

SWE of the surrounding liver parenchyma should be considered in patients with an IFLL, primarily to exclude underlying liver cirrhosis and for further risk stratification [50,58]. Owing to its availability on most up to date US scanners, it can be performed at the time of the diagnostic US.

**Quantitative ultrasound (QUS),** which includes a number of emerging ultrasound techniques, has shown promising results in characterizing FLLs. For instance, ultrasound tissue scatterer distribution imaging presented an overall AUROC of 0.81 in differentiating benign from malignant lesions [59].

**Combined imaging criteria using Fusion Imaging**. For IFLLs, most interventional diagnostic and treatment procedures are performed, at least in Europe, under US guidance. However, not all IFLLs are well seen on B-mode US. In order to avoid missing the target in these cases, fusion techniques have been developed. Basically, real-time US images move in synchronization with a previously performed CE-CT or a CE-MRI dataset. Thus, Fusion Imaging allows more accurate targeting of the IFLL for diagnostic (or biopsy) or therapeutic (radiofrequency ablation) purposes [60,61].

### 4.2. Computed Tomography (CT) and Contrast-Enhanced Computed Tomography (CE-CT)

CE-CT is the standard imaging method for staging malignant diseases, which is reflected in all generally accepted guidelines. CT is associated with radiation exposure, and the iodinated CAs used can, occasionally but rarely, lead to allergic reactions and renal failure. Radiation induced cancers have been identified as a major risk, particularly in younger patients in whom multiple follow-up CTs have been performed [62,63]. Thus, the use of CE-CT should be limited in young patients and avoided in those with a history of allergic reactions as well as in those with advanced chronic kidney disease. CE-CT should not be recommended for characterization of probable benign lesions, especially if equally accurate alternatives are available, owing to its limited accuracy, radiation exposure [64,65].

*Detection of IFLLs by Computed Tomography*. Published studies report that IFLLs are detected by CT in 7.2–33% of all patients investigated [27,28,66]. A more recent study, published in 2017, which included 17,309 participants who underwent screening CT for lung cancer, showed that potentially significant hepatobiliary changes were found in 2.1% of cases, 0.05% (*n* = 8) being liver cancers [29]. For the differential diagnosis of IFLLs, CE-CT with all vascular phases (arterial, portal-venous, and late) must be performed. However, this is not the case in all CT examinations. For example, if a patient undergoes an unenhanced thoracic scan that reveals an IFLL in the liver, this is not sufficient to fully characterize it, and thus it should be further investigated, taking into consideration the clinical context of the patient as well as other laboratory and imaging findings, even including CEUS, or CT/MRI in all vascular phases.

### 4.3. Contrast-Enhanced Magnetic Resonance Imaging (CE-MRI)

CE-MRI is a highly accurate imaging technique for the detection and characterization of IFLLs without ionizing radiation and with much fewer adverse events linked to the gadolinium-based contrast agents as compared to CE-CT [67]. CE-MRI utilizes two types of contrast agents. The first type, gadolinium-based CAs, have similar contrast enhancement patterns to those used in CE-CT. The second type is hepato-biliary specific, meaning that the CA (Gadoxetate disodium and Gadobenate diglumine) are taken up by the hepatocytes and excreted into bile, usually leading to better characterization and detection of IFLLs compared to non-liver-specific gadolinium-based contrast studies and CE-CT [68]. Gadolinium-based contrast agents used for CE-MRI have been associated with a risk of nephrogenic systemic fibrosis (NSF), but current newer macrocyclic agents are not [69]. However, CE-MRI is much more expensive than CE-CT, less available, and sometimes may not be possible owing to contraindications and claustrophobia [70]. MRI is also susceptible to motion artifacts, especially in the elderly, since long examination times and breath-holds are required. The presence of metallic or electrical implants can be a contraindication for MRI [36,37]. There is a lack on published real world data on how often MRI examinations are not possible or when the results are inconclusive.

*Detection of IFLL by Magnetic Resonance imaging*. The reported incidence of IFLLs found during breast MRIs ranged from 6 to 28% [30]. In one study, it was reported that follow-up investigations were recommended for 37.3% of IFLLs found during breast MRIs [31], but this number could be reduced to 5% using more specific recommendations and guidelines. Furthermore, in a recently published study, in which IFLLs were found in 11.3% of the 2181 assessed patients [29], abdominal radiology specialists agreed with the recommendation for follow-up imaging made by breast radiology specialists in only 7% of cases. This underlines the importance of gastroenterology/hepatology review and of establishing best practice liver imaging guidelines for IFLLs in order to reduce the number of unnecessary investigations.

## 5. Imaging (US)-Guided Biopsy, What Is the Importance of Histology?

The histological analysis of a representative pathological specimen, including immunohistochemistry, molecular analysis and other ancillary techniques allowing a precise definitive diagnosis, is recommended as a standard of care in most guidelines, prior to any neoadjuvant or palliative treatment [58,71,72,73]. However, owing to the development and accuracy of current imaging techniques, biopsies are less frequently needed for determining the nature of IFLLs [11,74]. In a German multicenter study, which included 1349 FLLs, only 6.8% of FLLs could not definitively be diagnosed using CEUS [75].

*Accuracy*. The diagnostic accuracy of percutaneous imaging-guided biopsy is approximately 90% [76,77]. There is a lack of published studies utilizing both state-of-the-art CE-CT and CEUS techniques owing to ethical issues. In small FLLs, <10 mm, the false-negative rate of percutaneous image-guided biopsy can be as high as 30% [78]. The accuracy of US-guided biopsies can be improved using CEUS guidance, which sometimes enables a better visualization of small nodules and avoids biopsy of necrotic areas [79,80,81]. In a recent randomized multicenter study, which included 2056 participants with FLLs, the accuracy of biopsy was significantly higher with CEUS-guidance compared to B-mode US-guidance only, especially in FLLs ≤ 20 mm and HCCs [82].

*Adverse events, needle tract seeding*. US-guided biopsy is a safe method, with significant hemorrhage occurring in approximately 0.5% of cases [71,83,84,85]. The rate of seeding following image-guided biopsy of a malignant liver tumor is not entirely negligible but is dependent on the histological type.

## 6. Clinical Significance of IFLLs

**Figure 1:**
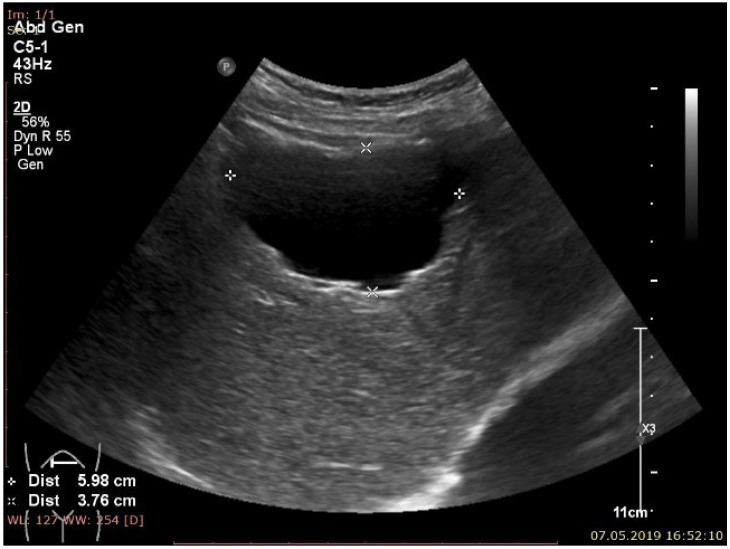
A 35-year-old male presents for consultation for nausea and diarrhea with acute onset. Ultrasound revealed a large, anechoic lesion (between markers x and +) with thin, irregular walls, situated in segment 4–5—typical aspect of simple biliary cyst.

**Figure 2:**
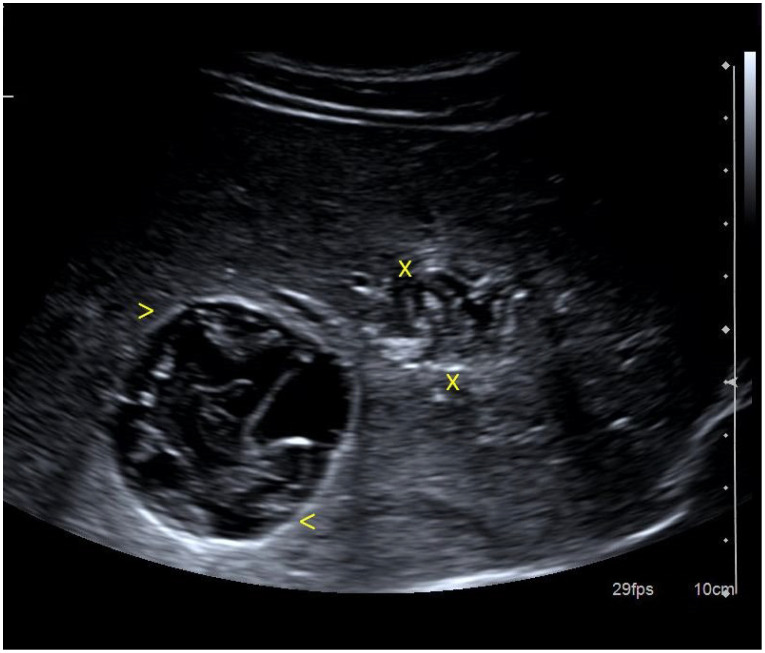
A 40-year-old female presents for consultation for right renal colic. Ultrasound revealed 2 cystic lesions (between markers x, and <) with thick walls and septa, situated in the right liver lobe. Anti Echinococcus granulosis antibodies positive. Typical aspect of hydatid cyst.

**Figure 3:**
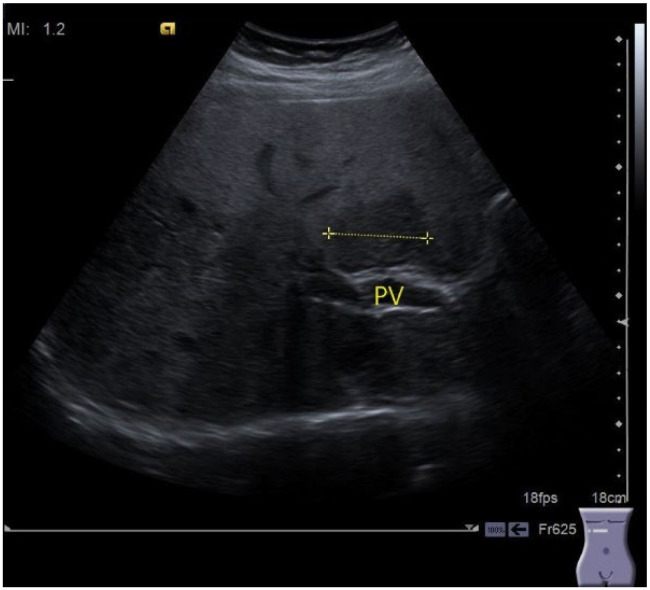
A 43 year-old obese female (BMI 32 kg/m^2^) presents for consultation for routine US examination. Ultrasound revealed a large hypoechoic area in segments VII, VIII with clear linear delineation from the rest of the liver. Just anterior to the portal vein (PV) another hypoechoic clearly delineated lesion (between markers +). Liver function tests normal, elevated triglycerides and glycemia, normal values of liver stiffness by 2D-SWE elastography. Typical aspect of focal fatty sparing.

**Figure 4:**
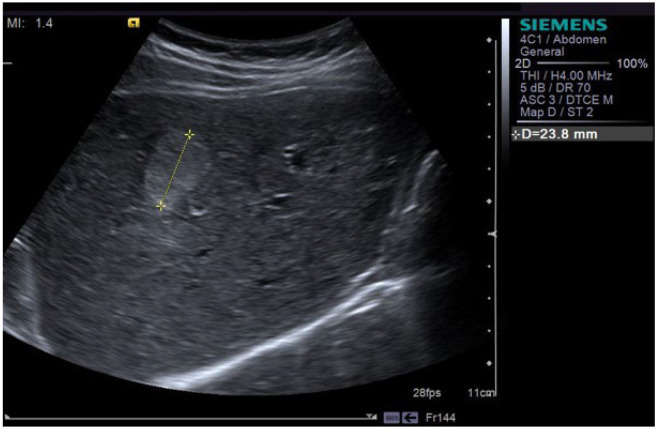
A 32-year-old male presents for consultation for occasional epigastric pain. Ultrasound revealed a hyperechoic, homogeneous, well delineated lesion (between markers +) 23 mm in diameter, situated in segment V—aspect of typical hemangioma.

*Is it benign or malignant?* The first question that needs to be answered when an IFLL is discovered by US is whether it is benign or malignant. More often, B-mode US alone cannot answer this question, except for the diagnosis of simple cysts (Figure 1 and Figure 2), classical focal fatty infiltration or focal fatty sparing (Figure 3) and typical hemangiomas (Figure 4).

Doppler imaging techniques may not really help the characterization of IFLLs, since they have a relatively low sensitivity and specificity and can be susceptible to artifacts [86,87,88]. However, Color Doppler Imaging and in particular Microvascular Doppler techniques should always be used to identify IFLLs with a typical and diagnosis-proving vascular pattern, in particular focal nodular hyperplasia (FNH), with a spoke wheel pattern [87].

There is constant debate on which contrast-enhanced imaging modality to use for the characterization of an IFLL. Over the last few years, CEUS has become the preferred method, especially for clinicians performing US. The advantage of CEUS is that it can be performed immediately after a standard US examination, considering relevant patient information, usually in approximately 5–10 min, utilizing the same US scanner. It is reassuring for patients when a newly diagnosed liver lesion can be immediately characterized without the need to wait for a CT or MRI scan. There are several pivotal studies including over 1000 FLLs and demonstrating that CEUS is an accurate method for the definitive diagnosis of FLLs [40,48], and in particular for differentiation between benign and malignant lesions. There are also published meta-analyses that demonstrate CEUS’s performance to be similar to that of CE-CT and CE-MRI [46,47]. In addition to a comparison of accuracy between contrast-enhanced imaging modalities, one must consider the problem of the costs and availability of CE-CT and CE-MRI, the waiting time to schedule such an investigation, and their possible drawbacks.

Considering all of the above and the fact that there are rigorous guidelines regarding the use of CEUS in clinical practice, updated several times [39,43,89,90], we advocate the use of CEUS as the first-line CE imaging method for the characterization of an IFLL where necessary. In this way, a definitive diagnosis can be reached rapidly in most cases. In cases of IFLLs which cannot be characterized sufficiently using CEUS, CE-CT has a very limited role. We suggest performing CE-MRI using a liver-specific contrast agent as the next step.

However, when performing CEUS, one must also bear in mind that the limitations of US also apply. Erroneous diagnoses by CEUS are most often linked to an inexperienced operator and incorrect instrument settings (use of a too-high mechanical index, inappropriate gain setting, inappropriate focus setting). Premature destruction of the microbubbles owing to an excessively high mechanical index or to continuous sonication of the lesion can mimic “washout” and lead to a false diagnosis of malignancy. Furthermore, if the examination is concluded prematurely, late “washout” can be missed, leading to a false diagnosis of a benign FLL.

In most cases, CE imaging methods are sufficient for characterizing IFLLs. However, some may remain indeterminate, thus necessitating a biopsy.

*Is malignant transformation possible in benign IFLLs?* In asymptomatic patients with a healthy liver, benign lesions such as hepatocellular adenoma (HCA), cholangiocellular adenoma and hepatic mucinous cystic neoplasms (formerly known as cystadenomas) have the potential for malignant transformation [39,91,92,93,94,95,96,97,98,99] and thus a scheduled surveillance or surgical treatment should be initiated based on systematic risk assessment [58,100,101].

*The asymptomatic patient.* First of all, a rigorous anamnesis and clinical exam, as well as lab tests, should be performed in order to ensure that the patient is truly healthy and asymptomatic. If anamnesis and laboratory tests reveal that there are potential risks associated with the patients, then a more aggressive diagnostic strategy and treatment options are indicated, taking into account the risk stratification, prognosis and clinical settings [74]. The latter is beyond the remit of this review, which concentrates on IFLLs in low-risk patients.

## 7. Ultrasound Features of IFLL

### 7.1. Size, Shape, Delineation

**Figure 5:**
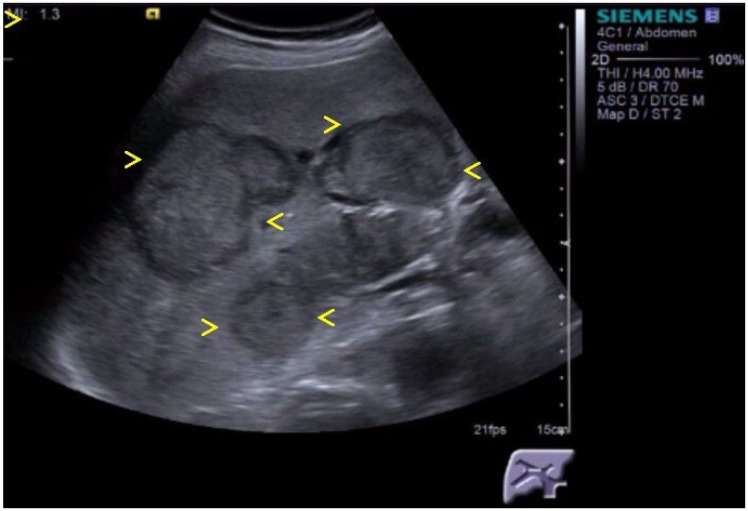
Several echogenic FLL with a hypoechoic peripheral rim “halo sign” (between arrows)—typical for metastases, in a 68-year-old patient with a history of colonic cancer.

Small lesions detected in asymptomatic patients have a very high (>95%) probability of being benign [26]. The shape is also important. Rounded, hypoechoic IFLLs are more frequently true solid lesions, while those with an angular shape or geographical delineation are more frequently related to fatty changes or perfusional abnormalities (“pseudolesions”). Benign liver tumors are usually well delineated, while malignant ones, in particular metastases, often depict the “halo sign”—a hypoechoic ring surrounding the lesion [102] (Figure 5).

A poorly delineated hypoechoic mass, with anechoic content, with thick, irregular walls and septa, in a patient with a fever, leukocytosis and a poor general state should be highly suspicious for a liver abscess. Regarding cystic lesions, the wall outline and thickness aid the differential diagnosis. For example, thin irregular walls are characteristic for simple benign biliary cysts, while thick walls can be seen with infections such as echinococcosis. Typical findings for the rare hepatic mucinous cystic neoplasms are unifocal cystic lesions with loculations, septations and cyst-in-cyst-appearance. Septations often are central and typically not associated to the external wall indentations. Septations and walls show contrast enhancement on CEUS, while (irregular) thickening of the wall or septae, contrast-enhancing mural nodules and associated segmental intrahepatic bile duct dilatation are highly sensitive and moderately specific for malignancy. Intraductal papillary neoplasm of the bile duct (PNBs) typically present as multifocal cystic mucin-containing dilatations of intrahepatic bile ducts, sometimes with contrast-enhancing papillary nodules [103,104,105,106,107]. Focal fatty changes do not have a mass effect on adjacent structure, invade vessels or alter the liver surface. Both benign and malignant liver tumors, as well as cystic lesions, can have a mass effect on adjacent vessels and alter the liver surface. Signs of vascular invasion are typical for malignant lesions.

### 7.2. Echogenicity in Comparison to the Surrounding Liver Parenchyma

The echogenicity of an FLL is a relative term and depends on the surrounding liver parenchyma. Thus, a hemangioma may be hyperechoic in a normal liver, but hypoechoic in steatosis hepatis. A focal nodular hyperplasia (FNH) may be isoechoic in a normal liver, but hypoechoic in a steatotic liver [39]. Echogenicity may be truly isoechoic to the surrounding liver parenchyma, but other situations have to be taken into consideration. In very small FLLs, the echogenicity cannot be detected by current transducer technology, which is true, for example, in von Meyenburg complexes (biliary hamartomas) [108]. These hamartomas may not be visible using the standard abdominal transducer, but higher frequency transducers may show the hyperechoic foci (“cholangiofibroma”) and small cystic parts of the lesions [109]. In addition, depending on the frequency of the transducer, FLLs may show up as isoechoic on standard abdominal transducers, but are hypoechoic with higher frequency transducers.

#### 7.2.1. Hyperechoic FLLs

Hemangiomas are the most frequently encountered hyperechoic and benign solid IFLLs, which can be found in 5–7% of patients. The classical appearance is of homogeneously hyperechoic, well delineated round or oval, usually smaller than 3 cm, solitary (70%) or multiple (30%) lesions. In a steatotic liver, hemangiomas can be hypoechoic, with posterior enhancement and hyperechoic borders. The “mirror effect” (a form of multipath artifact, in which a mirroring image of the FLL appears on the other side of the visceral pleura/diaphragm owing to the highly reflective echo at the interface between the liver surface and the visceral pleura/diaphragm) may be present. Larger hemangiomas are usually more inhomogeneous, owing to different histological content and/or secondary thrombosis, fibrosis and necrosis. The positive diagnosis in typical hemangiomas can be reliably made on B-mode US in most patients, if truly asymptomatic. If there is any doubt or atypical features are present, including a diameter > 3 cm, (approximately 30% of cases), CEUS should be used as the first-line CE imaging method [39], since its accuracy for diagnosing a benign FLL in healthy parenchyma is almost 100% while for the characterization of hemangioma it is about 92% [40]. Different types of hemangiomas have been described. The typical arterial phase enhancement pattern for hemangiomas is peripheral nodular contrast enhancement, with often slowly evolving centripetal “fill-in” complemented with hyperenhancement in the portal and late phases [39]. Beware of continuous insolation that can lead to pseudo-washout secondary to bubble destruction.

*Differential diagnosis*. On B-mode US, hemangiomas can be mistaken for focal fatty infiltration, but the latter often have typical locations (e.g., near the portal bifurcation, falciform ligament) and are usually angular or geographical in shape. On CEUS, focal fatty infiltration usually shows isoenhancement to the adjacent liver parenchyma in all vascular phases [39]. In approximately 10% of the cases, very small hemangiomas (<15 mm) show rapid arterial enhancement owing to the presence of arterio-portal shunts (“arterio-portal shunt-hemangioma”, “high-flow hemangioma”) [110], thus leading to problems distinguishing it from other benign FLLs, especially very small focal nodular hyperplasia (FNH). They are usually found in areas with focal fatty changes. CEUS can help differentiate echogenic malignant lesions, commonly metastases from neuroendocrine tumors or colonic carcinomas, which may also be hyperechoic, smoothly confined and hypervascularized on arterial hyperenhancement [39]. However, these malignant lesions will usually demonstrate washout in the portal-venous and late phases.

#### 7.2.2. Isoechoic FLL

Benign isoechoic IFLLs are FNHs or hepatocellular adenomas (HCAs), but hepatocellular carcinomas (more frequently occurring in a cirrhotic liver) and metastases can occasionally be isoechoic to the normal liver parenchyma [111].

*Differential diagnosis.* A diagnosis cannot be made without a contrast-enhanced imaging method, and sometimes, a US-guided biopsy is needed for the definitive diagnosis. This is mainly true for suspected metastases and to allow molecular typing. Typically, in CEUS, malignant lesions show washout in the portal-venous and late phases, this being the main criterion for differentiating them from benign lesions, which will often show iso- or hyperenhancement in all phases, if not cystic [39]. In low-risk patients, the main difficulty is differentiating between FNHs and hepatocellular adenomas (HCAs). Both show arterial hyperenhancement, FNHs with a centrifugal, “spoke wheel” pattern, while in HCAs, the filling is centripetal. FNHs remain hyperenhanced in all vascular phases in about 95% of cases, while HCAs do not contain portal veins and therefore should typically show mild washout in the portal-venous and late phases [39].

In accordance with the overlapping features of the subtypes and also the rarity of HCAs, the behavior of HCAs in the portal-venous and late phases may be variable. Inflammatory HCAs may typically exhibit arterial hyperenhancement with centripetal filling, peripheral rim enhancement, which may persist and central washout in the late venous phase. Twelve percent of all inflammatory hepatocellular adenomas (HCAs) still showed hyperenhancement in the late phase in CEUS, which was not observed in the other subtypes. It is assumed that there is an overlap between teleangiectatic FNHs and inflammatory HCAs [112]. In contrast, isoenhancement or moderate hyperenhancement with mixed filling in the arterial phase, and isoenhancement in the portal and late portal-venous phases, was observed in HNF1α-inactivated HCA [88,93]. As indicated, a definitive diagnosis may be difficult in a substantial number of cases [99,113,114,115]. Regarding the further differential diagnoses in the non-cirrhotic liver, we refer to the current WFUMB CEUS liver guidelines and comments [39,88,99,116,117,118,119].

#### 7.2.3. Hypoechoic Focal Liver Lesions

In a steatotic liver, an oval, hypoechoic lesion directly located at the liver hilum with a typical vascular supply on Color Doppler Imaging is most probably an area of focal fatty sparing (FFS). In rare cases, focal fatty sparing can mask an underlying FLL that will only be apparent with CE imaging [120]. Small so-called “shunt hemangiomas” should also be considered. These are masked by and typically located within an area of focal fatty sparing. In addition, FNHs and (much less likely) HCAs are also possible differential diagnoses. Ultimately, contrast-enhanced imaging is needed for characterization since hypoechoic lesions in a normal liver can be malignant, particularly when they display indistinct borders and the “halo sign” [39].

*Differential diagnosis*. Hypoechoic FLLs should be further characterized, especially if not typical for focal fatty sparing, in an otherwise steatotic liver. A FLL can be categorized as benign when it is isoenhanced or hyperenhanced in the late phase of CEUS, except in a cirrhotic liver [39]. On CEUS, in the arterial phase, a feeding artery can sometimes be visualized within the area of focal fatty sparing. However, it will be isoenhancing in all vascular phases [39].

The arterial phase of CEUS is very important for further characterization of benign FLLs, since some FLLs have typical enhancement patterns (specifically FNHs) allowing their definitive diagnosis. The main characteristic of malignant lesions in CEUS is that they do not retain contrast in the late phase. The only exceptions are a few usually well-differentiated HCCs in the cirrhotic liver and neuroendocrine metastases, which may not demonstrate any washout or show very late and mild hypoenhancement [121,122].

## 8. Evidence Based Recommendation

WFUMB and EFSUMB guidelines recommend CEUS as the first-line contrast imaging method for the characterization of IFLLs discovered by US in a non-cirrhotic liver and without a history of malignancy, as well as the second-line modality in patients with inconclusive CT or MRI findings [39].

The EASL (European Association for the Study of the Liver) guidelines on the management of incidentally found benign liver lesions, report nearly 100% specificity on all three CE imaging modalities for the diagnosis of FNHs and recommend the use of CEUS for the confirmation of suspected FNHs that are indeterminate on CE-MRI [58]. However, guidelines from the ACG (American College of Gastroenterology) and SBH (Brazilian Society of Hepatology) recommend only CE-CT and CE-MRI for establishing this diagnosis [74,123]. The reality in everyday life is that MRI highlights typical features of FNHs, but fails to differentiate a large number of arterially well-vascularized lesions, and these patients are often referred for a CEUS or biopsy.

MRI has been recommended for differentiating FNHs from HCAs, as well as for subtyping of HCAs, according to the guidelines from EASL, ACG and SBH [58,74,123]. In cases of doubt, EASL recommends a percutaneous liver biopsy [58]. Specific features on MRI have been described for the two most common subtypes of hepatocellular adenomas. Inflammatory hepatocellular adenomas have different patterns representing sinusoidal dilatation, including the newly described “sickle sign” [124]. HNF1α-mutated hepatocellular adenomas exhibit fat and a hypovascular pattern on MRI [124]. Both subtypes can be better assigned based on their enhancement characteristics on magnetic resonance imaging (MRI) with gadoxetic acid (Gd-EOB) [125]. Since imaging features do not reflect all molecular based alterations and data are sparse and not completely coherent, an accurate imaging-based differentiation of all HCA subtypes is not possible yet, even in specialized centers.

The recommendations of other scientific societies also differ regarding the diagnosis of hemangiomas. Whereas the 2016 published guidelines of EASL recommend contrast-enhanced imaging modalities only for patients with atypical hemangiomas and in patients with underlying oncological or liver disease, and refrain from prioritizing one of the three modalities (CEUS, CE-CT, CE-MRI) [58], the American College of Gastroenterology [74] and the Brazilian Society of Hepatology [123] Guidelines recommend only CE-CT and CE-MRI. The Appropriateness Criteria^®^ of the American College of Radiologists state that CEUS may be appropriate for the characterization of an indeterminate IFLLs > 1 cm found by non-contrast or single-phase CT or non-contrast MRI in a normal liver. In contrast to EASL, ACG and SBH discourage percutaneous biopsy, mainly due to a risk of bleeding [74,123]. Some of these disagreements between guidelines are related to differences in the availability of UCAs, issues related to the characteristics of the various health systems, and differing experience of using CEUS in different regions of the world.

## 9. Prognosis and Follow-Up

When a liver hemangioma is diagnosed based on specific imaging features, no follow-up or treatment is needed [58,74]. No follow-up is needed also for focal fatty change. When FNH is diagnosed based on specific imaging features, no follow-up or treatment is required [74]. Contraceptive use and pregnancy are not contraindicated according to ACG and EASL recommendations [58,74]. The ACG also does not exclude the use of anabolic steroids, while the EASL does not explicitly mention this. The ACG recommends follow-up for 2–3 years if contraceptives are taken. In the absence of contraceptive use, no follow-up is recommended. EASL generally does not recommend follow-up unless vascular liver disease is causative for FNH.

## 10. Surgery and Other Treatment Options

Liver hemangiomas and FNHs have a very low risk of bleeding and practically no risk of malignant transformation. Thus, there is generally no indication for surgery, with the very few exceptions of very large lesions that become symptomatic, causing recurring pain or compression [74]. The differential diagnoses of hemangiomas and hemangioendotheliomas should be mentioned, with washout on contrast imaging in the latter. Very rare treatment options for large FNHs have been described, including embolization or radiofrequency ablation [74].

Large hepatocellular adenomas in women (>5 cm in diameter) are considered to be at risk for spontaneous rupture (11–29% of cases) [74,126] or malignant transformation (5–10% of cases) [74,127]. In such cases, as well as in growing lesions, surgical resection is indicated.

Once an HCA is diagnosed in women, immediate cessation of oral contraceptives is indicated (if appropriate) [98]. Considering the relatively high risk of malignant transformation of HCAs in men, surgical resection is indicated in all HCAs diagnosed in men [58]. Another therapeutic approach is embolization in patients with active bleeding, high risk for surgery or if the lesion is in a difficult location [58,74]. Percutaneous biopsy should be considered for molecular diagnosis in cases of inconclusive findings [58,74,123]. The more specific diagnosis and treatment of HCAs, HCCs and metastases are beyond the remit of this review.

## 11. Rare Focal Liver Lesions

The imaging description of specific FLLs has been extensively described in the literature. We refer to the EFSUMB/WFUMB guidelines [39] and often cited published papers on the characterization of hemangiomas, focal nodular hyperplasias [40,128], hepatocellular adenomas [8,99,115,129,130,131,132], cholangiocellular adenomas, and hepatocellular carcinoma using the liver imaging reporting and data system (LI-RADS) [133,134,135], HCCs in the non-cirrhotic liver [108], fibrolamellar hepatocellular carcinomas [136], cholangiocellular carcinomas, and mixed HCC and cholangiocellular carcinoma and the specific features of very small HCC (<10 mm) [137].

We refer also to currently published papers supported by histological gold-standard for characterization of peliosis [138,139], hemangioendothelioma, nodular regenerative hyperplasia, sarcoma, inflammatory pseudotumor, sarcoidosis [140], tuberculosis, hydatid cysts [141], alveolar echinococcosis, schistosomiasis [142], ascariasis, fasciolosis, clonorchis and opisthorchis, toxocariasis, bacillary angiomatosis and amyloidosis with spontaneous hemorrhage, as well as rare FLLs in pediatric patients. Most recently rare bacterial [117], parasitic [119], and autoimmune focal liver lesions [118], bile duct lesions, other benign FLLs [88], and FLLs other than HCCs in the cirrhotic liver have been featured as well [117,118,119,120,121,122,123,124,125,126,127,128,129].

## 12. Challenges and Burdens Related to Incidental Detection of FLLs and Economic Issues

General ethical dilemmas, psychological burdens, the potential morbidity of further imaging and follow-up, and the extra cost-related issues have been discussed elsewhere and are not repeated here [1,6]. To name only a few: an accurate diagnosis of IFLLs is critical, not only to reassure patients with benign lesions but more importantly to correctly diagnose malignant lesions, thus avoiding the devastating consequences of a missed diagnosis and delayed treatment. The psychological burden for the patient is also not negligible, considering the long waiting time for cross-sectional imaging (CT/MRI), the side effects of contrast agents, and the ionizing radiation in CT, which can potentially have nefarious consequences for patients.

## 13. Conclusions

IFLLs are frequently found in clinical practice during liver imaging. In low-risk patients they are benign in most cases. In low-risk patients, if the IFLL has the typical imaging characteristics of a benign lesion when viewed by B-mode US, no further imaging is needed. CEUS should be used as the initial contrast-enhanced imaging technique to differentiate benign from malignant IFLLs in patients where there is any doubt, which has similar accuracy to CE-MRI. CE-CT should be avoided for characterizing probable benign FLLs and reserved for staging in known malignancy. In high-risk patients (significant chronic liver disease or an oncological history), each IFLL should be considered as potentially malignant, and no effort should be spared in order to confirm or exclude malignancy and make a specific diagnosis.

## Figures and Tables

**Table 1:** Frequency of incidentally found focal liver lesions.

Reference	Method	Number of Patients	IFLL, Including Fatty Liver	Benign FLL	Malignant FLL	Comment
Kaltenbach T et al. [22]	US	45.319	Not reported	15.1%	Not reported	Hospitalized patients
Rungsinaporn K, Phaisakamas T [14]	US	3.398	42% Fatty liver 35.6% Liver masses 6.2% patients	10.9% IFLL (372/3398)	3.4% IFFLL (7/3398)	Population study
Lu SN et al. [12]	US	923	27.5%	2.3%	Not reported	Population study
Hirche TO et al. [23].	US	255	19%	Not reported	Not reported	Pts. with Crohn’s disease
Choi SH [24]	US	2670	681 IFLL in 542 people	674 IFLL (99%)	7 IFLL (1.0%) in 3 people (0.6%)	Asymptomatic population
Ren Y, et al. [25]	US	21,629 (general) 17,721 (chronic hepatitis B)	12.5% (general) 40.6% (chronic hepatitis B)	12.5% (general) 40.6% (chronic hepatitis B)	Not reported	Further evaluation of IFLLs on CE-CT, CE-MRI and/or pathology
Little JM et al. [26]	CT + US	64	96.9%	79.7%	17.2%	All pts. with incidentalomas detected by CT or US, some of them with underlying chronic liver disease
Devine AS et al. [27]	CT	922	8.8%	7.5%		Pts. evaluated for trauma. IFLL not reported as benign or malignant
Volk M et al. [28]	CT	100	Not reported	33%	Not reported	Only benign IFLLs reported
Nguyen XV et al. [29]	CT	17.309	2.1%	Not reported	0.05%	Patients screened for lung cancer by low dose lung CT, potentially significant abnormalities reported
DiPiro PJ et al. [30]	MRI	2.181	11.3%	Not reported	Not reported	Patients evaluated by breast MRI
Knox M et al. [31]	MRI	1.664	12.4%	9.2%	0.9%	Patients evaluated by breast MRI
Galvao B et al. [32]	MRI	218	Not reported	13.8% cysts, 8.5% hemangiomas	Not reported	MRI-study: 218 cases with non—cirrhotic livers: 13.8% cysts, 8.5% hemangiomas, no other benign lesions.

**Table 2:** “Red flag” criteria (clinical and anamnestic data, hepatic parenchymal changes).

“Red Flag” Criteria	Which Liver Lesion Should Be Thought of Preferentially?
Old age	With increasing age, FLLs such as cysts and hemangiomas are more common [22]. But malignant tumors also occur more frequently with increasing age and can metastasize to the liver
Laboratory parameters (e.g., anemia); increase in tumor markers in relation to therapy	If the risk of tumors is high, FLLs may represent metastases
Clinical symptoms that may indicate malignancy (weight loss, cachexia, night sweats, specific clues such as blood in stool, palpable resistances)	FLLs may correspond to primary malignant liver tumors or metastases
Fever, high laboratory inflammatory parameters and abdominal inflammatory or infectious disease	Liver lesions may represent abscesses or inflammatory pseudotumors
Liver cirrhosis or chronic liver disease with advanced fibrosis	There is an increased risk of hepatocellular carcinoma (HCC). 76–81% of all newly diagnosed FLL in liver cirrhosis corresponded to HCC [38]
Tumor history, either with a treated malignant tumor in the past or with a currently diagnosed malignant disease	In connection with a tumor history, any FLL is initially suspicious for a metastasis until proven otherwise. Some tumors can still metastasize after a very long interval (e.g., breast cancer)

## Data Availability

Not applicable.
